# Supplementary material for: An Amidase Contributes to Full Virulence of Sclerotinia sclerotiorum
Source: Int J Mol Sci. 2022 Sep 23;23(19):11207. doi: 10.3390/ijms231911207 (PMC9570306; doi:10.3390/ijms231911207)
Supplement: Supplementary file 1 [file ijms-23-11207-s001.zip › ijms-1899947-supplementary.pdf]

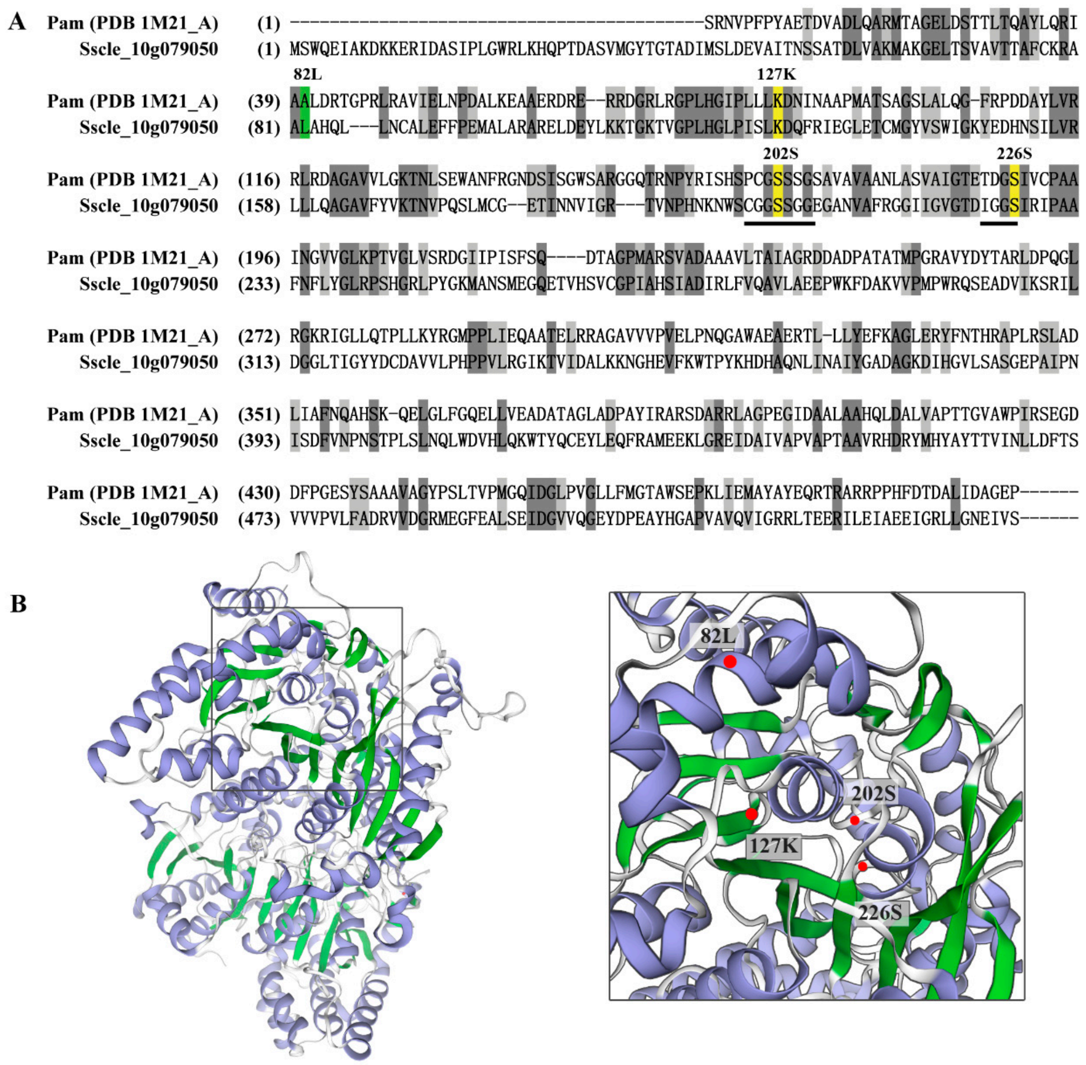

Supplementary Figure S1. (A) Structural sequence alignment of amidase signature (AS) region of Pam from *S. maltophilia* (PDB ID: 1M21) and Sscle_10g079050 protein. The Gly/Ser-rich sequence and oxyanion hole are underlined with black lines and the Ser-*cis* Ser-Lys catalytic triad is indicated by yellow highlight. (B) The overall 3D-structure of Sscle_10g079050 protein predicted by SWISS-MODEL using a fatty acid amide hydrolase (FAAH) as template. The catalytic triad Ser^{202}-Ser^{226}-Lys^{127} and Leu82 are labeled by red dots.

Supplementary Table S1 Primers in this study

Primer name	Primer sequence	Primer function
KO 1F	5' GGCTTCATCACCATCGTTATCAG 3'	KO 1F and KO 2R for amplification of upstream sequence of *Sscle_10g079050*.The lowercase part presents homologous sequences from HY fragment using for homologous recombination.
KO 2R	5' ccttcaatatcatcttctgCAGATAGGGTGCCACGACA 3'	
KO 3F	5' gtttgaggtaatccttcTTTAGATTTGCTGTGATGACTTGAG 3'	KO 3F and KO 4R for amplification of downstream sequence of *Sscle_10g079050*. The lowercase part presents homologous sequences from YG fragment using for homologous recombination.
KO 4R	5' CGAGGGGATGGGTGGAATGTTTA 3'	
PF	5' CAGGGGAGAGATGAATGCTGACAC 3'	PF and PR for real transformants identification
PR	5' CGACCACCTTCCTCTCAGACAT 3'	
Seq F	5' AGTATTCCTCTGGGATGGCG 3'	Seq F and Seq R for sanger sequencing
Seq R	5' GAAGAACCACCGCAAGACCAG 3'	
HYG-F	5' CAGAAGATGATATTGAAGGAGCAC 3'	HYG-F and HY-R for amplification of HY fragment
HY-R	5' GCATCATCGAAATTGCCGTCAACC 3'	
YG-F	5' TCTCGGAGGGCGAAGAATCTCGTGC 3'	YG-F and HYG-R for amplification of YG fragment
HYG-R	5' AAAGAAGGATTACCTCTAAACAAGTGT 3'	
KI-PR	5' ccttcaatatcatcttctgAGAAACAATCTCATTCCCA 3'	KO 1F and KI-PR for amplification of upstream sequence and genomic DNA of *Sscle_10g079050*. The lowercase part presents homologous sequences from HY fragment using for homologous recombination.
TF	5' GCTCCGTAACACCCAATACGCCG 3'	TF and PR for downstream sequences confirmation in transformants.
HY-PR	5' CGCCGCCGCTACTGCTACAAGTG 3'	PF and HY-PR for upstream sequences / genomic DNA sequences confirmation in transformants.
